# Comparison of Secondary Prevention Following Hysteroscopic Adhesiolysis in the Improvement of Reproductive Outcomes: A Retrospective Cohort Study

**DOI:** 10.3390/jcm13010073

**Published:** 2023-12-22

**Authors:** Tianyu Wu, Tao Fang, Yuanhang Dong, Jingxia Mao, Jia Wang, Ming Zhao, Ruijin Wu

**Affiliations:** Department of Gynecology, Women’s Hospital, School of Medicine, Zhejiang University, Hangzhou 310006, China

**Keywords:** intrauterine adhesion, intrauterine devices, hyaluronic acid gel, hysteroscopic adhesiolysis, pregnancy rates

## Abstract

Intrauterine adhesion (IUA) is primarily caused by endometrial injury, and hysteroscopic adhesiolysis is presently the main treatment. However, postoperative recurrence and poor pregnancy outcomes remain intractable. In this study, we aim to assess the effects of different treatments on clinical symptoms and reproductive outcomes in IUA. This retrospective study was conducted in a tertiary university-affiliated women’s hospital. The study included 1449 consecutive women who desired to have a baby and were diagnosed with IUA through hysteroscopy from January 2016 to December 2021. Patients with IUA underwent hysteroscopic electric resection (E) or cold scissors separation (C), as well as hormone therapy and one or both of the following secondary prevention measures: intrauterine devices (IUD) and hyaluronic acid gel (HA). The pregnancy rate (PR) was significantly higher in the E + IUD + HA (90.23% CI: 85.82, 94.64%) than in other groups (*p* = 0.000) groups. The rates of full-term birth (*p* = 0.000) and live birth (*p* = 0.000) were significantly higher in the E + IUD + HA (67.82% and 68.97%, respectively) and E + HA (62.41% and 63.91%, respectively) groups. Multivariate logistic regression analysis revealed a significantly higher PR in women who received second-look hysteroscopy (OR 1.571, 95% CI: 1.009–2.224, *p* = 0.013) and E + IUD + HA (OR 4.772, 95% CI: 2.534–8.987, *p* = 0.000). Combining hysteroscopic electric resection with IUDs and HA gel could prevent adhesion recurrence and improve postoperative pregnancy and live birth outcomes in IUA. Furthermore, postoperative second-look hysteroscopy may increase the PR and shorten the waiting period.

## 1. Introduction

Intrauterine adhesion (IUA) is a condition characterized by partial or complete adhesion between the anterior and posterior walls of the uterine cavity after an endometrial injury, always involving the basal layer of the endometrium, and induced by infection, inflammation, miscarriage, dilatation, curettage, and other operations [1]. Its prevalence varies widely by the type of intrauterine procedure that causes the damage; for instance, women who have undergone postpartum curettage exhibit a prevalence as high as 21.5% [2]. The condition can be asymptomatic but commonly results in hypomenorrhea or amenorrhea, infertility, and recurrent pregnancy loss, which severely affect women’s physical and mental well-being [2].

Hysteroscopic adhesiolysis is the most widely used treatment for IUA, which involves both energy-based methods using electrodes and mechanical methods using cold scissors. Electrodes convert electricity into heat to achieve local cutting or electrocoagulation for hemostasis and separation, but they may also cause thermal injury to the endometrium and result in some complications, including miscarriages during midterm pregnancy [3]. The cold-scissor approach is supposed to prevent thermal damage to the residual endometrium and reduce the risk of perforation during the procedure. However, it is unfavorable for stopping the bleeding and may, therefore, be unsuitable for patients with moderate and severe adhesion [4]. Overall, there is no consensus on which hysteroscopic method is preferable.

With all current therapies, adhesion is surgically detached. Nevertheless, the detachment operation could lead to re-adhesion, as new wound formation can cause inflammatory exudation and collagen hyperplasia. A study reported that the rate of re-adhesion after hysteroscopic separation for IUA was as high as 62.5% in severe patients [5]. Therefore, several modalities have been studied and suggested for preventing new adhesion, including intrauterine devices (IUDs) and hyaluronic acid (HA) gel. IUD placement has long been commonly used for maintaining the uterine cavity [6]. It provides a mechanical barrier between the uterine walls and separates the wound surfaces during the initial healing phase, diminishing the likelihood of re-adhesion [7]. Conversely, it also tends to cause intrauterine excessive inflammatory response or infection, incarceration, and perforation. Hyaluronic acid, a biopolymer composed of repeating units of disaccharides, which belongs to mucopolysaccharides of glycosaminoglycan’s family, is widely applied for preventing re-adhesion [8]. The excellent absorbability and histocompatibility of HA aids in separating the tissue surface during wound repair, thereby preventing the formation and adhesion of fibrous tissue [9]. Moreover, estrogen has also been suggested as a perioperative adjuvant therapy to improve prognosis and prevent recurrent adhesion [10]. Several studies have shown that estrogen can promote endometrial growth, as it exhibits a significant increase in angiogenesis and a significant decrease in fibrosis [11]. However, recent studies have reported that higher dosages have no superior effect over lower dosages (10, 8, and 6 mg compared with 4 mg, and 6 mg compared with 2 mg); thus, the side effects of high-dosage hormone therapy cannot be ignored [12,13,14]. Consequently, the IUA treatment remains confronted with great challenges.

Additional research has been required because of the lack of a definitive best therapy. The present study is designed to find a more effective strategy and provide doctors and patients with more valuable guidance on surgical and secondary treatments. The main variable for this analysis was, therefore, the type of treatment. We also explored the protective and risk factors associated with the reproductive prognosis of patients with IUA.

## 2. Materials and Methods

### 2.1. Ethics Statement

The study was approved by the Ethics Committee of Women’s Hospital, Zhejiang University School of Medicine (No. IRB-20220259-R).

### 2.2. Patients

Patients were recruited from Women’s Hospital, Zhejiang University, from January 2016 to December 2021. The inclusion criteria included the following: (1) women aged ≥20 and ≤40 years old with a desire to have a baby; (2) diagnosed with IUA through hysteroscope. The exclusion criteria included the following: (1) prescribed hormones for at least 3 months before the first surgery and prescribed hysteroscopic adhesiolysis for at least 6 months before the first surgery; (2) other factors affecting fertility, such as diminished ovarian reserve, endometriosis, adenomyosis, and chronic salpingitis; (3) reproductive malformation and serious reproductive system diseases including uterus septum and malignant tumor; (4) pathological diagnosis of other lesions, including endometrial polyp, cervical canal polyp, and submucosal leiomyoma, after the first hysteroscopic surgery during the follow-up period; (5) lost to follow-up.

### 2.3. Evaluation

Before treatment, all patients had undergone conventional preoperative evaluations. These evaluations included a detailed history of age, body mass index (BMI), menstrual pattern, previous intrauterine operation, reproductive history (gestation and parturition), and transvaginal ultrasonography.

The severity and extent of IUA were scored according to the American Fertility Society (AFS) classification for IUA [15]. Baseline and posttreatment AFS scores were evaluated using hysteroscopy. A score of 1–4 represents mild adhesion, a score of 5–8 represents moderate adhesion, and a score of 9–12 represents severe adhesion.

### 2.4. Interventions

The procedure settings were decided upon the initiation of the case. All operative hysteroscopy was performed by 1 of 2 reproductive surgeons at our hospital using the same techniques. Hysteroscopy was performed using a 5-French hysteroscope (KARL STORZ GmbH & Co. KG, Tuttlingen, Germany) with a monopolar energy source. Glucose 5% (Guojing Pharmaceutical Co., Ltd., Lishui, China) solution was administered at an electronically controlled flow rate to distend the uterine cavity and assist in removing adhesion. Surgeons dissected the adhesion using a hook-shaped electrode or cold scissor, depending on the specific circumstances. Electrode resection was performed with 10-mm conventional resectoscopes, and cold scissor separation was performed with 5-mm mini-resectoscopes. Based on the surgeon’s observation, women received one of the following secondary adhesion preventions after surgery: (1) insertion of a Copper Yuan Gong containing indomethacin IUD (Yuangongyao Copper 200, Yantai JiShengYaoXie Co., Ltd., Yantai, China); (2) intrauterine administration of 3 mL sodium hyaluronate gel (Gongankang, BioRegen Biomedical Co., Ltd., Changzhou, China); or (3) both Yuangongyao Copper 200 and 3 mL sodium hyaluronate gel. The 2 surgeons taking part in this study were rigorously and similarly trained and assessed, and they had been conducting this kind of operation for over 5 years.

After hysteroscopy, hormone therapy was administered as follows: a daily dosage of 4 mg of estrogen (Progynova, Jenapharm GmbH & Co. KG, Roubaix, France) for 21 days, followed by a daily intake of 20 mg of dydrogesterone (Duphaston, Abbott Healthcare Products B.V., Houtenlaan, The Netherlands) for the final 10 days of the estrogen therapy. The hormone therapy was repeated for 2 to 4 cycles based on the surgeon’s observations during the procedure. Eight patients underwent only 1 cycle of hormones due to certain side effects. Twelve patients underwent 5 or 6 cycles of hormone therapy, depending on their condition’s severity or personal preference.

Overall, patients with IUA underwent hysteroscopic electric resection (E) or cold scissors separation (C), as well as hormone therapy and one or both of the following secondary prevention measures: intrauterine devices (IUD) and hyaluronic acid gel (HA).

The second hysteroscopy would be performed if the patient had diminished blood loss over time following the successful removal of the adhesion and abdominal pain or if the ultrasonography findings indicated uneven endometrial echoes. In addition to assessing the extent and severity of any reformed adhesion, hysteroscopic adhesiolysis was also conducted during the second-look procedure for patients experiencing recurrent adhesion.

### 2.5. Follow-Ups

Follow-ups were performed either in the clinic or by telephone consultation, ending in June 2022. The following information was recorded at the first follow-up 3 months after intrauterine surgery: operative complications and endometrial thickness. At the same time, the IUD would be removed. The decision of second-look hysteroscopy was based on ultrasonography and clinical manifestations. We evaluated AFS scores among the women who received a second look. Active attempts at conception could be made if the assessment indicated that the endometrial condition had improved. Afterward, follow-ups were performed in the clinic or by telephone consultation every 6 months to 1 year until the pregnancy was achieved. During subsequent follow-ups, we investigated postoperative menstrual patterns and pregnancy outcomes, including the date of the first pregnancy, term delivery, live birth, abnormal pregnancy results (miscarriage and preterm birth), and pregnancy complications (premature rupture of membranes). The primary outcome of our study was clinical pregnancy, which was defined as a pregnancy diagnosed by ultrasound visualization of one or more gestational sacs. Secondary outcomes included endometrial thickness, posttreatment menstrual patterns, postoperative AFS scores, term delivery, live birth, miscarriage, preterm birth, and premature rupture of membranes.

### 2.6. Statistical Analysis

Proportions for categorical variables and means (±standard deviation) for continuous variables depending on their normal distribution were used for descriptive purposes. The confidence level of proportions for categorical variables was 95%. Categorical variables were compared with the chi-square test. Fisher’s exact test was used instead of the chi-squared test when the value of a categorical variable was <5. Ordered multi-categorical variables were compared with the Kruskal–Wallis H test. Continuous variables with a normal distribution were compared with the independent *t*-test, differences among multiple groups were tested using analysis of variance, and *p*-values of multiple comparisons were adjusted using Bonferroni’s adjustment. Multivariate logistic regression analysis was performed to identify factors that affected the pregnancy rates (PR). Time-related cumulative pregnancy rates were compared in the Kaplan–Meier model and a log-rank test was performed. Data were excluded when the sample size was less than or equal to 1. *p* < 0.05 was considered statistically significant. All statistical analysis was performed using IBM SPSS Statistics for Windows (Version 25.0., IBM Corp., Armonk, NY, USA).

## 3. Results

**Figure 1:**
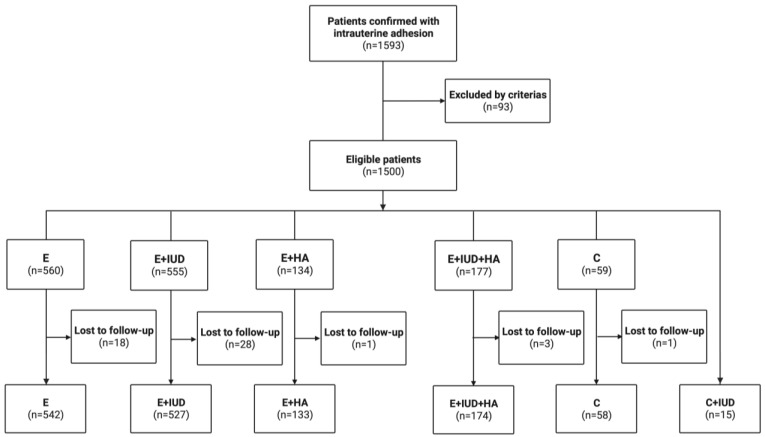
Flowchart of patients’ enrollment.

The recruitment process is shown in Figure 1. Of the 1449 women included in the final analysis, 542 received E and hormone therapy (E group), 527 received E, IUD, and hormone therapy (E + IUD group), 133 received E, HA, and hormone therapy (E + HA group), 174 received E, IUD, HA, and hormone therapy (E + IUD + HA group), 58 received C and hormone therapy (C group), and 15 received C, IUD, and hormone therapy (C + IUD group). A discrete subgroup underwent second-look hysteroscopic evaluation and treatment (586/1449, 40.44%), while the remaining participants did not. The baseline characteristics of these women before the treatments are presented in Table 1. Among the groups, age, BMI, reproductive history, preoperative endometrial thickness, AFS scores, and adhesion extent were comparable (*p* > 0.05). There were significant differences in the times of any previous intrauterine operations (*p* = 0.001) and the rates of the second hysteroscopy (*p* = 0.000). Specifically, women in the E + IUD + HA group had undergone more intrauterine operations compared with other groups; the rate of second looks in the E group (32.84%) was lower than that in the E + IUD (43.64%) and E + IUD + HA (48.28%) groups. The patients who underwent a second hysteroscopy were more prone to displaying symptoms and ultrasound after the initial hysteroscopic adhesiolysis.

The results of the comparison of clinical characteristics and reproductive outcomes among different groups are shown in Table 2. There were significant differences in menstrual improvement (*p* = 0.000), growth rates of endometrial thickness (*p* = 0.000), drop rates of AFS score (*p* = 0.000), and posttreatment AFS scores (*p* = 0.000) among the groups. After the treatments, there were significant improvements in menstrual patterns and AFS scores in the E + HA and E + IUD + HA groups compared with other groups. The growth rates of endometrial thickness were higher in the E and E + IUD + HA groups than those in the E + IUD and C + IUD groups.

The follow-up of the 1449 patients lasted for 6–60 months. The PR was significantly higher in the E + IUD + HA (90.23% CI: 85.82, 94.64%) than in the E (73.05% CI: 69.32, 76.80%) and E + IUD (76.82% CI: 73.25, 80.45%) groups (*p* = 0.000). Additionally, the miscarriage rate was significantly lower in the E + IUD + HA (13.79%) and E + HA (12.78%) groups (*p* = 0.000). The rates of full-term birth were higher in the E + IUD + HA (67.82%) and E + HA (62.41%) groups (*p* = 0.000). The live birth rate (LBR) in the E + IUD + HA (68.97% CI: 62.10, 75.84%) and E + HA (63.91% CI 55.71, 72.07%) groups were higher than that in the others (*p* = 0.000). However, the rate of premature rupture of membranes was higher in the E + IUD + HA group (40.23%) (*p* = 0.000). The preterm rates were comparable among all groups (*p* > 0.05). Altogether, patients in the E + IUD + HA and E + HA groups might gain relatively more reproductive benefits from their treatments.

**Figure 2:**
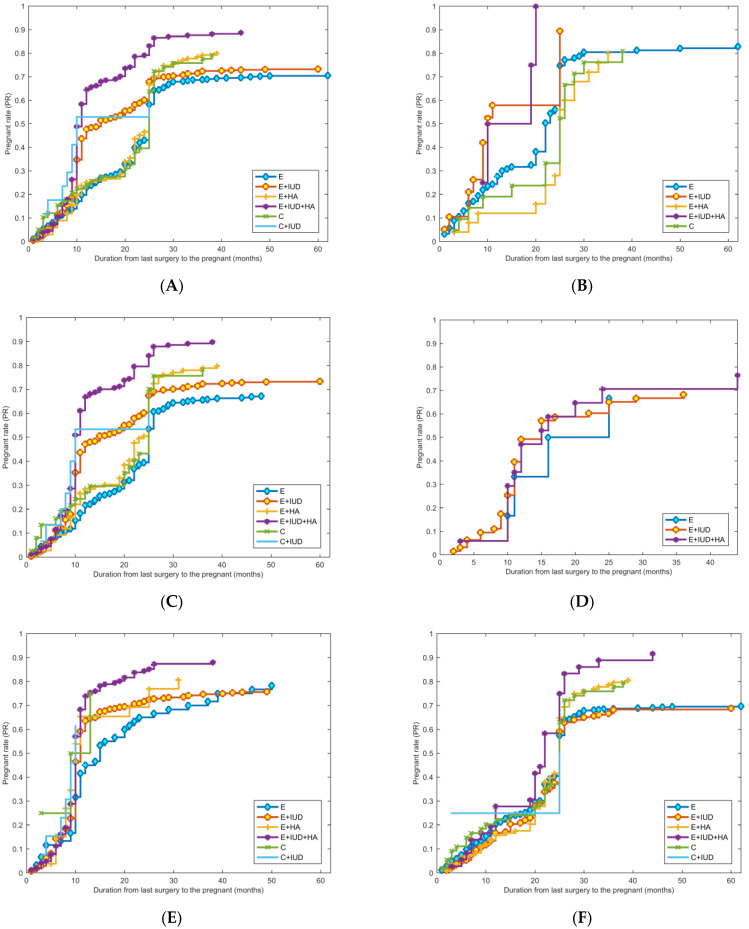
Kaplan–Meier plot for treatments associated with PR. (**A**) Kaplan–Meier plot for treatments associated with PR in all patients with IUA, *p* < 0.003; (**B**–**D**) Kaplan–Meier plot for treatments associated with PR in patients with mild (*p* < 0.005), moderate (*p* < 0.003), and severe (*p* < 0.016) adhesion, respectively. (**E**,**F**) Kaplan–Meier plot for treatments associated with PR in patients who received second looks (*p* < 0.05) and who did not (*p* < 0.05).

For further analysis, we constructed a time-related cumulative PR curve of different treatments in the Kaplan–Meier model. We set the month of the last hysteroscopy of the treatment procedure as month 0 and counted the months from the last surgery to the pregnancy date. The PR dramatically increased in the first 2 years after the operation and tended to stabilize after 2 years. The E + IUD + HA group showed a significantly higher PR than the other groups (*p* < 0.003). As fertility declined with ascending severity and extent of IUA, we compared postoperative reproductive outcomes among patients with mild, moderate, and severe adhesion. Regardless of the severity and extent of IUA, the PR in the E + IUD + HA group was significantly higher than that in the other groups (*p* < 0.005, *p* < 0.003, *p* < 0.016 in mild, moderate, and severe, respectively). Simultaneously, the PR was significantly higher in the E + IUD + HA group than in the other groups, whether or not the second hysteroscopy was employed. These Kaplan–Meier plots are shown in Figure 2.

A total of 1123 patients (77.5%) became pregnant after the treatment, among which 1029 (91.63%) spontaneously became pregnant, and 94 (8.37%) became pregnant by assisted reproductive technology (ART). We then compared some factors associated with reproductive outcomes between the pregnant and non-pregnant groups; the results are shown in Table 3. Multivariate logistic regression analysis revealed that single endometrial thickness ≥0.35 cm (OR 1.996, 95% CI: 1.105–3.604, *p* = 0.022) and second-look hysteroscopy (OR 1.571, 95% CI: 1.009–2.224, *p* = 0.013) were protective factors for reproductive outcomes of patients with IUA. Patients aged >35 years (OR 0.120, 95% CI: 0.070–0.206, *p* = 0.000), moderate (OR 0.462,95% CI: 0.283–0.755, *p* = 0.002), and severe adhesion (OR 0.415, 95% CI: 0.196–0.897, *p* = 0.022) were factors significant to patient-related risk factors associated with the reproductive outcomes of fertility-desiring women with IUA. Different treatments were significantly related to the pregnancy outcomes (*p* = 0.000). Compared with the treatment of the E group, the treatments of the E + IUD (OR 1.479, 95% CI: 1.014–2.156, *p* = 0.042), E + HA (OR 2.121, 95% CI: 1.248–3.604, *p* = 0.005), and E + IUD + HA (OR 4.772, 95% CI: 2.534–8.987, *p* = 0.000) groups showed gradually increasing positive effects on reproductive outcomes. The C and C + IUD groups showed no significant difference compared with the E group. Reproductive history and previous intrauterine operations showed no significant relationship with the reproductive outcomes (*p* > 0.05).

**Figure 3:**
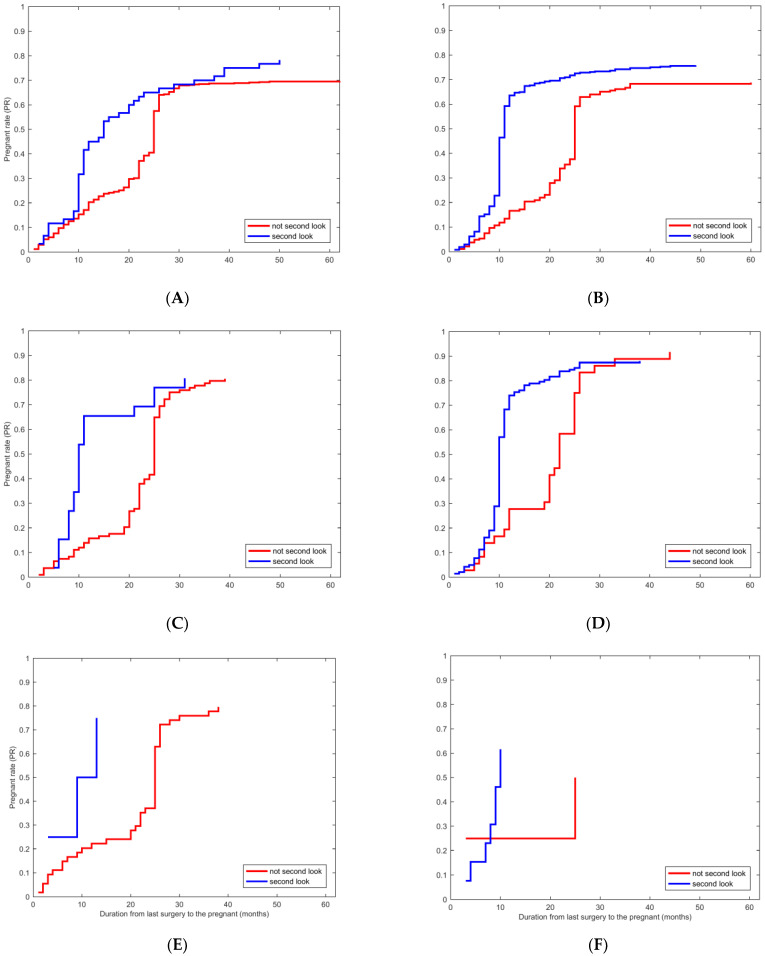
Kaplan–Meier plot for the second look associated with PR in patients who received different treatments. (**A**–**F**) Kaplan–Meier plot for the second look associated with PR in patients in different treatment groups, the E group (*p* < 0.05), the E + IUD group (*p* < 0.05), the E + HA group (*p* < 0.05), the E + IUD + HA group, the C group, the C + IUD group, respectively.

Additionally, we compared the PR among patients who received second looks and those who did not across different treatment groups. As displayed in Figure 3, the PR was significantly higher in patients who underwent second-look hysteroscopic examination than those who did not in the E (*p* < 0.05), E + IUD group (*p* < 0.05), and E + HA (*p* < 0.05) groups. Moreover, the duration between the last surgery and the first pregnancy was shorter among patients who received second-look hysteroscopy across all groups (*p* < 0.05) except the C group (Table 4). Second-look hysteroscopy was a significantly positive factor for reproductive outcomes for fertility-desiring patients.

## 4. Discussion

IUA has become one of the main diseases seriously affecting women’s reproductive health owing to its annual increase. An ideal treatment for IUA would completely remove adhesion and restore the shape and volume of the uterine cavity in preparation for a subsequent pregnancy [1]. To our knowledge, only a few reports have compared currently used treatments with no standard clinical pathway. The present study contained a large cohort of women with IUA and follow-ups of their reproductive outcomes after adhesiolysis, including live birth, which was an essential desire of an infertile couple.

In our study, the menstrual patterns, the AFS scores, the PR, and the LBR were better in the E + HA and E + IUD + HA groups than in other groups. The multivariate analysis showed that the treatments of E + IUD + HA had better effects on PRs. In summary, the E + IUD + HA treatment was more beneficial in preventing re-adhesion and promoting endometrial growth, followed by the E + HA treatment. This might be attributed to their barrier function, separating the wound surfaces. However, the variations in the treatment effectiveness were relatively less prominent in severe patients, though significant, which might be attributed to their heightened severity of endometrial damage and decreased receptivity. Trinh et al. reported that a combination of HA gel and IUDs provided more excellent prevention of recurrent IUA and decreased posttreatment AFS scores for infertile women undergoing hysteroscopic adhesiolysis; however, the ongoing PR after in vitro fertilization did not improve [16]. A recent meta-analysis also demonstrated that combining HA gel and IUDs was the most effective strategy for reducing AFS scores and IUA severity, whereas hysteroscopic administration of HA gel was associated with a higher PR [17]. Additionally, in studies where estrogen was used ancillary with IUDs, the PRs were generally higher than those in the studies where estrogen was used alone [18]. Additional research is required on prior treatments for enhancing pregnancy outcomes based on large-scale studies.

Notably, we noticed a higher incidence of premature rupture of membranes in the E + IUD + HA group, which might be related to the greater number of intrauterine operations performed on the patients in this group. Intrauterine manipulation has the potential to cause endometrium and myometrium injury with varying degrees and damage to the internal cervical sphincter, which could expose the membranes to the vagina, thereby affecting their strength and integrity [19]. However, the reasons for the high rate of premature rupture of membranes remained unclear.

As for C and E treatments, a recent study reported no remarkable difference in the PRs of hysteroscopic cold knives and hysteroscopic electroacupuncture. However, hysteroscopic cold knife separation for IUA had less influence on patients’ endometrium, indicated by increased menstrual flow and decreased AFS scores. The cold knife could remove adhesive tissue more thoroughly as it was equipped with miniature surgical scissors and could be inserted at 3 mm under direct vision to cut off the adhesive tissue in the uterus [20]. Except for the scar management, another possible reason for the higher AFS scores of electrodes was that the energy-based equipment might cause damage to the endometrium by increasing inflammatory cytokines and promoting adhesion-causing agents such as transforming growth factor-beta, platelet-derived growth factor, and fibroblast growth factor after the operation. A randomized, prospective study revealed that the mini-resectoscope exhibited comparable performances to the conventional resectoscope concerning menstrual and reproductive outcomes while significantly decreasing operative morbidity [21]. However, we did not observe any differences in adhesion prevention and pregnancy improvement between the treatments of electrode and cold scissor treatments nor between the treatments employing classical and mini-resectoscopes. Additional research is necessary to validate our findings because of the brief follow-up period and the small sample size. Furthermore, appropriate training and rigorous assessment are crucial for treatment effectiveness and safety, including holding endoscopic workshops and evaluations regularly [22].

Furthermore, the postoperative second look was an independent beneficial factor for the PR. It could even shorten the duration between the pregnancy date and the last intrauterine surgery, consistent with previous studies [7,23,24]. An early second look could decrease the local inflammation and prevent adhesion recurrence by washing away residual blood clots, necrotic tissue, and inflammatory factors in the uterine cavity; these factors subsequently affect clinical prognosis and reproductive outcomes. However, there was no agreement on the timing of the second-look hysteroscopy. According to previous studies on the healing process, weak fibrous adhesions initially held the wound together after adhesiolysis, and the wound tensile strength would increase 5 days later [25]. Di Spiezio Sardo et al. suggested that adhesion reformed during the first postoperative hours; thus, follow-up hysteroscopy on the second or third postoperative day might be useful [23]. Pabuccu et al. demonstrated that control hysteroscopy should be performed no later than 1 week after the initial procedure [7]. Xu et al., reported that early second-look hysteroscopic examinations within 2 months may increase the cumulative PR and LBR [24]. The adhesive process may be progressive; therefore, early intervention may be essential for prognosis. However, the timing of second-look hysteroscopy varied from 3 to 6 months in our study, warranting further research. Additionally, the average duration between the first pregnancy and the last operation was at least about 10 months in various treatment groups in the present study. Nevertheless, other studies have demonstrated that the optimal period for subsequent fertility treatment after hysteroscopic surgeries was 3–6 months [26,27]. The waiting period might be reduced if patients with IUA received ART after adhesiolysis or second hysteroscopic examinations within 3 months. We noticed that the rate of second look in the E group was lower than that in the E + IUD and E + IUD + HA groups, which might be attributed to the comparatively lower severity of IUA in the E group.

Besides the extent of adhesion, the treatments, and the second look, the age and the endometrial thickness also influenced the pregnancy outcomes. Age and fertility are closely related, as is the case in IUA patients after adhesiolysis. The older a patient is, the worse their ovarian reserve and the higher the incidence of chromosomal abnormalities during oocyte maturation, leading to poor pregnancy outcomes [28]. Our results demonstrated that patients would gain better pregnancy outcomes when the single endometrial thickness was at least 0.35 cm. Endometrial thickness is considered to be associated with endometrial receptivity. Thin and damaged endometrium loses receptivity and hampers implantation in women with IUA [29].

In obstetrics and gynecology, adhesion formation is a critical postoperative complication that may lead to chronic abdominal pain and infertility. A recent systemic review indicated that a range of physical barrier antiadhesive agents are promising for reducing adhesion formation effectively [30]. Several limitations exist concerning surgical adhesiolysis, antiadhesive agents, and hormonal therapy aimed at restoring the endometrium. Multipotent mesenchymal stromal cells (MMSCs), extracellular vesicles derived from MMSCs, as well as biological barriers, such as the amniotic membrane, small intestine submucosa, urinary bladder matrix, along with platelet-rich plasma (PRP), provide alternative methods for the management of IUA and other cases of adhesion [31,32]. Further studies are required to verify their impact on reproductive outcomes and to establish precise guidelines for their per-operative application.

Our investigation had some limitations. First, a discrete group did not undergo second-look hysteroscopic evaluation and treatment, and the rates of second-look were significantly different among the groups. Additionally, the patients who received a second hysteroscopy were likely to have exhibited more symptoms and ultrasound features after the first hysteroscopic adhesiolysis. Therefore, there might be some information biases. Furthermore, the decrease from pretreatment to posttreatment AFS scores may not equal the improvement of intrauterine status; thus, the decrease may not completely reflect the effect of anti-adhesion therapy [16]. Thus, we could not analyze the recurrence intuitively, which was the main factor affecting postoperative pregnancy and live birth [20]. Second, the pregnancy patterns following hysteroscopic adhesiolysis may contribute to different reproductive outcomes for IUA, so doctors are often faced with the challenge of deciding whether to facilitate spontaneous pregnancy or use ART [33]. However, we did not investigate the pregnancy patterns in baseline characteristics analysis; thus, some confounding bias might exist. Moreover, the types may be as important as the times of previous intrauterine operations. Unfortunately, we did not count the ratio of the different types of intrauterine operations, including dilatation and curettage, hysteroscopy, or hysteroscopic adhesiolysis, which might introduce a selection bias and pose a significant limitation. Dilatation and curettage pose risk factors for both intrauterine adhesion and premature delivery. Moreover, type 3 fibroids, a distinct subtype of intramural fibroids, represent a more special case that can potentially impact influencing pregnancy outcomes [34]. Hysteroscopic resection emerges as a potential alternative to conventional surgery for type 3 myomas, but further evidence is imperative to evaluate the safety and efficacy of hysteroscopic treatment for type 3 myomas [35,36]. In our study, few patients had undergone hysteroscopic resection of type 3 myoma. Furthermore, our study was based on a single-center retrospective analysis, which entailed inherent biases and a lack of generalizability.

## 5. Conclusions

In summary, our findings suggest that combining hysteroscopic electric resection, IUDs, HA gel, and hormone therapy could increase the probability of pregnancy and live birth. The postoperative second-look hysteroscopy was an independent protective factor for pregnancy and could shorten the duration between the first pregnancy and the last operation. An adequately powered randomized controlled trial is required to confirm our findings and conduct a more comprehensive evaluation of the combination therapy’s efficacy in preventing adhesion recurrence and promoting favorable reproductive outcomes among infertile women.

## Figures and Tables

**Table 1:** Baseline characteristics of included patients.

Characteristics	E (*n* = 542)	E + IUD (*n* = 527)	E + HA (*n* = 133)	E + IUD + HA (*n* = 174)	C (*n* = 58)	C + IUD (*n* = 15)	*p*-Value
Age (years)	30.43 ± 4.66	30.90 ± 4.90	30.99 ± 4.46	31.10 ± 4.64	30.56 ± 4.77	31.00 ± 5.05	0.479
BMI (kg/m^2^)	21.35 ± 2.94	21.33 ± 2.99	20.91 ± 2.43	21.69 ± 2.99	21.47 ± 2.83	21.03 ± 2.91	0.423
Gravidity	2.48 ± 1.39	2.74 ± 1.48	2.67 ± 1.48	2.84 ± 1.67	2.36 ± 1.35	2.79 ± 2.47	0.300
Parity	0.45 ± 0.57	0.48 ± 0.61	0.46 ± 0.56	0.44 ± 0.56	0.36 ± 0.55	0.35 ± 0.61	0.711
Times of any previous intrauterine operations	2.04 ± 1.33 a	2.14 ± 1.35 a	2.20 ± 1.41 a	2.82 ± 1.58 b	1.90 ± 1.25 a	2.36 ± 2.40 a	0.001 **
Preoperative endometrial thickness (single, cm)	0.28 ± 0.99	0.27 ± 0.08	0.26 ± 0.07	0.25 ± 0.07	0.26 ± 0.04	0.29 ± 0.06	0.124
Pretreatment AFS scores	5.42 ± 1.41	6.03 ± 1.54	5.61 ± 1.40	6.97 ± 1.50	4.73 ± 1.31	6.59 ± 1.54	0.086
Adhesive extent ^#^							0.3012
Mild	119 (21.96)	18 (3.42)	25 (18.80)	4 (2.30)	21 (36.21)	0 (0.00)	
Moderate	416 (76.75)	449 (85.20)	108 (81.20)	153 (87.93)	37 (63.79)	15 (100.00)	
Severe	7 (1.29)	60 (11.39)	0 (0.00)	17 (9.77)	0 (0.00)	0 (0.00)	
Second look	178 (32.84) a	230 (43.64) b	61 (45.86) ab	84 (48.28) b	25 (43.10) ab	8 (53.33) ab	0.000 **

Data are represented as means (SD) and proportions as appropriate. ** *p* < 0.01; ^#^ Kruskal–Wallis H test; The data with different little letters in the same line show significant differences. Abbreviations: BMI, body mass index; AFS, American Fertility Society.

**Table 2:** Comparison of reproductive outcomes among different groups.

Outcomes	E (*n* = 542)	E + IUD (*n* = 527)	E + HA (*n* = 133)	E + IUD + HA (*n* = 174)	C (*n* = 58)	C + IUD (*n* = 15)	*p*-Value
Menstrual improvement ^#^	a	b	cd	d	b	bcd	0.000 **
Little	273 (50.37)	59 (11.20)	7 (5.26)	10 (5.75)	15 (25.86)	1 (6.67)	
Slight	263 (48.52)	442 (83.87)	43 (32.33)	44 (25.29)	42 (72.41)	10 (66.67)	
Significant	6 (1.11)	26 (4.93)	83 (62.41)	120 (68.97)	1 (1.72)	4 (26.67)	
The growth rate of endometrial thickness	0.50 ± 0.85 a	0.11 ± 0.46 b	0.35 ± 0.48 ab	0.58 ± 0.58 a	0.45 ± 0.46 ab	0.11 ± 0.44 b	0.000 **
The decline rate of the AFS score	0.61 ± 0.24 a	0.62 ± 0.34 a	0.88 ± 0.29 b	0.93 ± 0.30 b	0.65 ± 0.17 a	0.63 ± 0.35 a	0.000 **
Posttreatment AFS scores	4.80 ± 1.61 a	4.11 ± 1.82 b	4.26 ± 1.69 abc	3.55 ± 1.73 c	4.00 ± 1.16 abc	3.23 ± 2.05 bc	0.000 **
Pregnancy	396 (73.06) a	405 (76.85) a	108 (81.20) ab	157 (90.23) b	47 (81.03) ab	10 (66.67) ab	0.000 **
Miscarriage	197 (36.35) a	88 (16.70) b	17 (12.78) b	24 (13.79) b	25 (43.10) a	4 (26.67) ab	0.000 **
Preterm	33 (6.09)	43 (8.16)	8 (6.02)	15 (8.62)	5 (8.62)	0 (0.00)	0.566
Premature rupture of membranes	42 (7.75) a	100 (18.98) b	28 (21.05) b	70 (40.23) c	6 (10.34) ab	2 (13.33) abc	0.000 **
Full-term birth	166 (30.63) a	274 (51.99) b	83 (62.41) bc	118 (67.82) c	17 (29.31) a	6 (40.00) abc	0.000 **
Live birth	199 (36.72) a	282 (53.51) b	85 (63.91) cd	120 (68.97) d	22 (37.93) a	6 (40.00) abc	0.000 **

Data are represented as means (SD) and proportions as appropriate. ** *p* < 0.01; ^#^ Kruskal–Wallis H test; The data with different little letters in the same line show significant differences.

**Table 3:** Factors associated with pregnancy rates by multivariate logistic regression analysis.

Variables		Adjusted OR (95% CI)	*p*-Value
Age (>35 years)		0.120 (0.070–0.206)	0.000 **
Endometrial thickness (≥0.35 cm, single)		1.996 (1.105–3.604)	0.022 *
Adhesion degree	Mild (Reference)		0.008 **
	Moderate	0.462 (0.283–0.755)	0.002
	Severe	0.415 (0.196–0.897)	0.022
Gravity	<2 times (Reference)		0.240
	2 times	0.671 (0.271–1.661)	0.389
	>2 times	0.428 (0.156–1.173)	0.099
Parity	<2 times (Reference)		0.940
	2 times	1.221 (0.402–3.713)	0.725
	>2 times	0.000 (0.000–)	1.000
Intrauterine operations	<2 times (Reference)		0.240
	2 times	2.039 (0.884–4.704)	0.095
	>2 times	1.813 (0.734–4.479)	0.197
Treatments	E group (Reference)		0.000 **
	E + IUD group	1.479 (1.014–2.156)	0.042
	E + HA group	2.121 (1.248–3.604)	0.005
	E + IUD + HA group	4.772 (2.534–8.987)	0.000
	C group	1.730 (0.818–3.659)	0.152
	C + IUD group	0.953 (0.279–3.255)	0.939
Second look		1.571 (1.009–2.224)	0.013 *

* *p* < 0.05, ** *p* < 0.01.

**Table 4:** Comparison of duration between first pregnancy and last surgery in the treatment procedure.

Treatment Groups	Duration between First Pregnancy and the Last Operation (Month)		*p*-Value
	Second look treated	Second look untreated	
E group	14.19 ± 7.37	21.26 ± 7.73	0.000 **
E + IUD group	11.76 ± 7.12	17.90 ± 10.17	0.000 **
E + HA group	12.65 ± 4.34	19.71 ± 2.73	0.000 **
E + IUD + HA group	11.42 ± 4.75	20.34 ± 7.87	0.000 **
C group	11.53 ± 8.20	16.18 ± 8.35	0.080
C + IUD group	8.75 ± 4.40	17.14 ± 7.82	0.022 *

Data are represented as means (SD). * *p* < 0.05, ** *p* < 0.01.

## Data Availability

Data were obtained from Women’s Hospital, School of Medicine, Zhejiang University. According to relevant regulations, the data cannot be shared but can be requested from the corresponding author.
